# HIV-Associated Mycobacterium tuberculosis Bloodstream Infection Is Underdiagnosed by Single Blood Culture

**DOI:** 10.1128/JCM.01914-17

**Published:** 2018-04-25

**Authors:** David A. Barr, Andrew D. Kerkhoff, Charlotte Schutz, Amy M. Ward, Gerry R. Davies, Robert J. Wilkinson, Graeme Meintjes

**Affiliations:** a Wellcome Liverpool Glasgow Centre for Global Health Research, Institute of Infection and Global Health, University of Liverpool, Liverpool, United Kingdom; b Wellcome Centre for Infectious Diseases Research in Africa, Institute of Infectious Disease and Molecular Medicine, Department of Medicine, University of Cape Town, Cape Town, South Africa; c Division of Infectious Disease, Department of Medicine, University of California San Francisco School of Medicine, San Francisco, California, USA; d Department of Clinical Infection, Microbiology and Immunology, Institute of Global Health, University of Liverpool, Liverpool, United Kingdom; e Department of Medicine, Imperial College London, London, United Kingdom; f Francis Crick Institute, London, United Kingdom; Virginia Commonwealth University Medical Center

**Keywords:** Mycobacterium tuberculosis, blood culture, human immunodeficiency virus

## Abstract

We assessed the additional diagnostic yield for Mycobacterium tuberculosis bloodstream infection (BSI) by doing more than one tuberculosis (TB) blood culture from HIV-infected inpatients. In a retrospective analysis of two cohorts based in Cape Town, South Africa, 72/99 (73%) patients with M. tuberculosis BSI were identified by the first of two blood cultures during the same admission, with 27/99 (27%; 95% confidence interval [CI], 18 to 36%) testing negative on the first culture but positive on the second. In a prospective evaluation of up to 6 blood cultures over 24 h, 9 of 14 (65%) patients with M. tuberculosis BSI had M. tuberculosis grow on their first blood culture; 3 more patients (21%) were identified by a second independent blood culture at the same time point, and the remaining 2 were diagnosed only on the 4th and 6th blood cultures. Additional blood cultures increase the yield for M. tuberculosis BSI, similar to what is reported for nonmycobacterial BSI.

## INTRODUCTION

Mycobacterium tuberculosis bloodstream infection (BSI) is a frequent and life-threatening presentation of tuberculosis in high-HIV-burden settings. Published cohorts of HIV-1-infected inpatients with suspected tuberculosis show a point prevalence ranging from 9% (1) to 38% (2) on a single blood culture. M. tuberculosis BSI has been associated with severe sepsis (2–5) and high risk of death (5–8) in people living with HIV.

Several methods for the recovery of mycobacteria from blood exist, including a manual solid-medium-based lysis centrifugation system (Wampole Isostat/Isolator microbial system; bioMérieux, Durham, NC, USA) and automated liquid-medium systems (MB BacT/Alert; Inverness, Waltham, MA, USA; and Bactec Myco/F Lytic; BD Microbiology Systems, Sparks, MD). Broth-based systems are probably more sensitive than agar (8, 9). Beyond this, there are limited data on how to optimize blood culture for the diagnosis of M. tuberculosis BSI.

In contrast, evidenced-based recommendations on the number, timing, and volume of blood cultures are available for nonmycobacterial BSI, where a single 10-ml blood culture will detect 73% and four samples will detect 90 to 95% of patients with documented bacteremia (10, 11). Almost all published studies of M. tuberculosis BSI have performed single 3- to 5-ml liquid mycobacterial cultures, and the proportion of M. tuberculosis BSI missed by this strategy is unknown. We estimated the diagnostic yield of additional (>1) blood cultures for M. tuberculosis in two ways: (i) retrospectively, in two large cohort studies of HIV-associated tuberculosis (TB) conducted in hospital settings; and (ii) in a prospective evaluation of serial blood cultures in HIV-infected patients at high risk of M. tuberculosis BSI in a hospital setting.

## MATERIALS AND METHODS

Ethical approval was granted by the Human Research Ethics Committee of the University of Cape Town (Ref 001/2012, Ref 057/2013, and 057/2013 amendment 24/04/2016). Both the cohort studies and the prospective evaluation were carried out in the Western Cape, South Africa, a setting in which HIV and TB are the most common causes of death among adults, despite a well-functioning antiretroviral program (12). The GF Jooste Hospital TB (JHTB) study recruited unselected HIV-infected patients newly admitted to acute medical services at GF Jooste Hospital without a known TB diagnosis and who were not on anti-TB therapy. These patients underwent extensive microbiological screening for TB, including a single 5-ml BD Bactec Myco/F Lytic blood culture (BD, Sparks, MD) on the day of admission (13). The Khayelitsha Hospital (KHTB) study recruited HIV-infected patients admitted with symptoms suggestive of active tuberculosis and a CD4 count of less than 350 cells/mm^3^ and also performed routine 3- to 5-ml Bactec Myco/F Lytic blood cultures prior to the start of anti-TB therapy (14). Both of these hospitals had access to mycobacterial blood culture investigations through the National Health Laboratory Service (NHLS). A subset of patients in both cohorts had additional M. tuberculosis blood cultures, which were requested by their admitting medical team if clinically indicated (local guidelines recommend TB blood culture if CD4 count is less than 100 cells/mm^3^ in a patient with TB symptoms where there is difficulty obtaining sputum samples for TB testing or the sputum Xpert MTB/RIF assay is negative, and cultures are generally sent before the start of anti-TB therapy). By interrogating the NHLS electronic database, we identified the subsets of patients in both cohorts who had second BD Bactec Myco/F Lytic blood cultures carried out as part of routine care during the same admission as their study recruitment.

To enrich recruitment to the prospective study of serial blood cultures, we used data from 350 KHTB patients to develop a model predicting M. tuberculosis BSI in patients using only the clinical variables available on the day of admission to Khayelitsha Hospital (15). This model used an ensemble machine learning approach combining logistic regression, random forest, and support vector machine methods and gave a receiver operator characteristic (ROC) curve area under the curve of 0.86 in a test data set comprising 66 KHTB patients not used in model training. This ensemble model was packaged in a web-based application available at the patient bedside via a smart phone (16).

Between 21 June 2016 and 19 October 2016, on the weekdays Monday through Thursday, all HIV-infected patients newly admitted to Khayelitsha Hospital with CD4 counts of <350 cells/μl and suspected TB that had not yet started on anti-TB therapy were screened using the M. tuberculosis BSI prediction app. Patients with predicted probabilities of greater than 0.56 who gave informed consent underwent 3 venesections over a 24-h period: immediately before (0 h), 4 to 8 h after, and 22 to 24 h after first dose of anti-TB therapy. At each of these venesections, 5 ml of peripheral blood was directly inoculated into a Myco/F Lytic Bactec (BD, Sparks, MD) bottle, while 5 ml was collected in a sodium heparin tube and immediately centrifuged for 25 min at 3,000 × *g*, and the resulting cell pellet (red cells and buffy coat) was inoculated into a Myco/F Lytic bottle. Samples were transported to an NHLS TB laboratory in Cape Town for incubation the same day. The isolate identity was confirmed in all cases by secondary Löwenstein–Jensen slope culture, auramine acid-fast microscopy, and PCR/line probe assay.

## RESULTS

Using data from two independent cohort studies—the GF Jooste Hospital TB (JHTB) study and the Khayelitsha Hospital TB (KHTB) study—we identified HIV-infected inpatients who had multiple mycobacterial blood cultures performed during a single admission to the hospital with suspected TB. More than one blood culture was recorded for 59/410 JHTB patients and 169/680 KHTB patients, giving a total sample size of 228 for analysis. Of these patients, 99/228 (43%) had at least one blood culture positive for M. tuberculosis (20/59 in JHTB and 79/169 in KHTB). Overall, 72/99 (0.73; 95% confidence interval [CI], 0.64 to 0.82) of M. tuberculosis BSIs were identified on the first culture, while 27/99 (0.27; 95% CI, 0.18 to 0.36) had a negative first culture but grew M. tuberculosis on the second (Table 1).

**TABLE 1:** Additional M. tuberculosis BSI diagnoses made by second Myco/F Lytic culture in KHTB and JHTB cohort studies

Cohort	No. of cases with:^*a*^				Proportion identified only by 2nd culture (95% CI)^*b*^
	Either culture positive	BC1+/BC2+	BC1+/BC2−	BC1−/BC2+	
KDHTB	79	44	17	18	0.23 (0.14–0.32)
JHTB	20	7	4	9	0.45 (0.23–0.67)
Combined	99	51	21	27	0.27 (0.18–0.36)

a BC1, 1st Myco/F Lytic blood culture; BC2, 2nd Myco/F Lytic blood culture; +, positive; − negative.

b CI, confidence interval by binomial distribution.

To further investigate the yield of additional mycobacterial blood cultures, we carried out a prospective evaluation of multiple blood cultures in 16 HIV-infected inpatients at Khayelitsha Hospital. On the basis of baseline clinical variables and a machine learning algorithm, these patients were selected to have a high predicted probability of M. tuberculosis BSI (see Materials and Methods). A set of 2 5-ml blood cultures were performed at time zero and 4 to 8 h and 22 to 24 h after the first dose of anti-TB medication, with a total of 6 cultures over a 24-h period. Because of the potential for antimicrobial carryover in blood, the second sample from each pair had the plasma removed before inoculation of centrifuge-pelleted cells.

**FIG 1:**
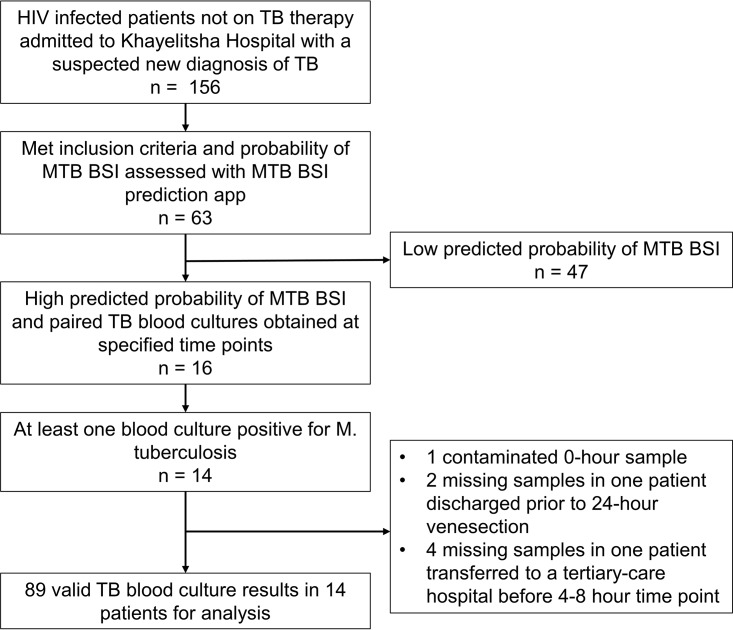
Patient recruitment and blood culture availability in prospective study. MTB BSI, M. tuberculosis bloodstream infection.

In total, 89 blood culture results were available from the 16 patients, with 7 results missing (Fig. 1). Of these, 32/89 (36%) were positive for M. tuberculosis. Pelleted samples were more likely to result in the recovery of M. tuberculosis (19/44 [43%]) than directly inoculated samples (13/45 [29%]), but the difference may have been due to chance (*P* = 0.189 by Fisher's exact test).

**FIG 2:**
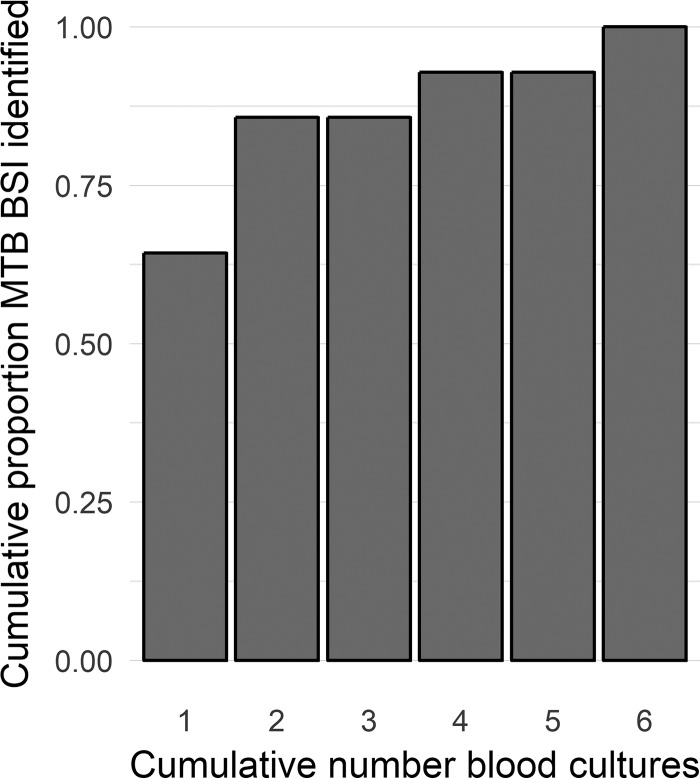
Cumulative yield for identifying M. tuberculosis BSI with up to 6 serial Myco/F Lytic blood cultures.

Two independent blood culture samples were obtained at each time point. At least one blood culture was positive in 14/16 (87.5%) patients. All isolates were identified as M. tuberculosis. Nine of fourteen (64%) of these patients were culture positive on the first sample from the pair of samples taken at time zero. A further 3/14 (21%) were culture negative on the first sample but grew M. tuberculosis on the second sample taken at the time zero time point. This meant that 12/14 (86%) of M. tuberculosis BSI patients were identified by performing 2 independent cultures at the same time point before antibiotic therapy. The remaining two patients were identified on the 4th and 6th blood cultures (both pelleted before inoculation) (Fig. 2).

## DISCUSSION

Using two independent data sets and a dedicated prospective evaluation, we estimate that approximately two-thirds of M. tuberculosis BSI is identified by one Myco/F Lytic blood culture (55% and 73% in the data sets, and 64% in the prospective evaluation). To our knowledge, this is the first investigation of the additional yield associated with the number of TB blood cultures. One previous study randomized patients for 6 blood cultures at a single time point or 3 blood cultures at 2 time points (but the same total number of cultures) and found no difference in the recoveries of M. tuberculosis between these arms (8). This agrees with our prospective study finding that two blood cultures at the same time point increase the yield compared to that from a single culture.

The importance of bloodstream infection in HIV-associated tuberculosis disease is increasingly recognized. “Disseminated” tuberculosis causes 2 of 5 inpatient deaths among HIV-infected inpatients in low-resource settings and is undiagnosed prior to death in half of these cases (17). This dissemination is assumed to occur via the bloodstream, and despite practical limitations, blood culture can be considered the gold-standard diagnostic test (18). TB blood culture positivity is associated with significantly higher mortality than blood culture-negative HIV-associated TB (5–8). In the context of a generalized HIV epidemic, M. tuberculosis is the most frequent blood culture isolate in hospitalized patients with severe sepsis (2–5).

With few exceptions, (7, 8) reports characterizing M. tuberculosis BSI have relied on a single blood culture for diagnosis; our results show this will have substantially underestimated the true point prevalence. This has implications for studies of HIV-associated TB pathogenesis and supports calls for increased clinical research focused on M. tuberculosis BSI, including the development of blood-based rapid diagnostics (6). Where resources currently allow, an additional TB blood culture will increase the culture diagnosis in seriously unwell HIV-infected inpatients, particularly when sputum is unobtainable.

Although several independent data sets have been used in this study, our findings are not generalizable outside high-HIV-TB-burden settings. Most high-HIV-TB-burden settings do not have routine access to TB blood cultures. However, the findings are useful to inform research studies carried out in those settings. In this study, we were unable to assess the relative cost-effectiveness of additional blood cultures compared to other diagnostics, such as induced sputum or urine Xpert MTB/RIF Ultra and the urine-lipoarabinomannan assay, which are potentially more accessible in low-resource settings. However, the data presented in this report give an opportunity to improve the reference standard in diagnostic performance studies assessing these novel diagnostics in the most critically unwell HIV-associated TB patients.

### Conclusion.

We estimate that a single TB blood culture underestimates the point prevalence of M. tuberculosis BSI by approximately one-third. Additional blood cultures—even within the same 24-h period—increase the diagnostic yield by a proportion similar to that seen for nonmycobacterial BSI. We recommend, where resources allow, that at least 2 blood cultures be taken when M. tuberculosis BSI is suspected in unwell HIV-infected adults, particularly when sputum is unobtainable. These can be collected at the same time point, prior to anti-TB treatment, in patients starting urgent empirical therapy.
